# Project EX: A Program of Empirical Research on Adolescent Tobacco Use Cessation

**DOI:** 10.1186/1617-9625-2-12

**Published:** 2004-09-15

**Authors:** Steve Sussman, William J McCuller, Hong Zheng, Yvonne M Pfingston, James Miyano, Clyde W Dent

**Affiliations:** 1 Institute for Health Promotion & Disease Prevention Research, University of Southern California, Alhambra, California, USA

## Abstract

This paper presents the Project EX research program. The historical background for Project EX is presented, including a brief summary of reasons youth fail to quit tobacco use, the disappointing status of previous cessation research, and the teen cessation trial that provided the template for the current project (Project TNT). Next, program development studies for Project EX are described. Through use of focus groups, a theme study (concept evaluation of written activity descriptions), a component study, and pilot studies, an eight-session program was developed. This program involves novel activities (e.g., "talk show enactments," games, and alternative medicine-type activities such as yoga and meditation) in combination with motivation enhancement and cognitive-behavioral strategies to motivate and instruct in cessation initiation and maintenance efforts. The outcomes of the first experimental trial of Project EX, a school-based clinic program, are described, followed by a posthoc analysis of its effects mediation. A second EX study, a multiple baseline single group pilot study design in Wuhan, China, is described next. Description of a second experimental trial follows, which tested EX with nicotine gum versus a natural herb. A third experimental trial that tests a classroom prevention/cessation version of EX is then introduced. Finally, the implications of this work are discussed. The intent-to-treat quit rate for Project EX is approximately 15% across studies, double that of a standard care comparison. Effects last up to a six-month post-program at regular and alternative high schools. Through a systematic protocol of empirical program development and field trials, an effective and replicable model teen tobacco use cessation program is established. Future cessation work might expand on this work.

## Introduction

Most regular adolescent tobacco users are likely to continue to use tobacco into adulthood [1]. These youth begin to suffer physical consequences of tobacco use within a year of regular use and are likely to become stereotyped as risky youth by nonsmokers. They generally try to quit tobacco use in adolescence, but find that they are unable to do so due to psychological dependence on tobacco and nicotine addiction. Approximately 40% of adolescent smokers who have smoked in the last month report having tried to quit (mostly self-initiated cessation) at some point in the past and failed [2,3]. While 53% of adolescent smokers report an interest in quitting tobacco use in the next six months, only approximately 18% of adolescent smokers are ready to take action and try to quit in the next 30 days [4]. Only 1% to 2% of heavy lifetime adolescent smokers (e.g., smoked greater than 100 cigarettes; now smoke 10 cigarettes per day or more) report self-initiated quitting for at least a 30-day period [3,5,6].

Generally, tobacco-related programmatic efforts have been focused on the development of teen tobacco use prevention programming or adult cessation programming. In fact, policy makers have begun to place a priority on teen tobacco use cessation research and practice only over the last 10 years. The Robert Wood Johnson Foundation, National Cancer Institute, National Institute on Drug Abuse, American Lung Association, and Health Canada, among numerous other agencies, have only recently aggressively promoted work in this arena. Up to this time, programs developed to facilitate teen tobacco use cessation have been few in number, most have been poor in research design, and most have been lacking or non-disclosing in program development details [2,3,7-9]. This paper describes a program of research focused on one adolescent tobacco use cessation project, Project EX, as an illustration of long-term systematic efforts to develop successful programming in this arena. This program of research began in 1987 as part of another research project and continues to the present time.

As a first step in this review of Project EX, we discuss reasons for quitting and not quitting tobacco use. In particular, the importance of motivating quit attempts to counteract barriers to quitting is emphasized. Next, the history of teen tobacco use cessation research is summarized. Then the history of Project EX is discussed, including its predecessor program, Project TNT, and the set of Project EX-1 program development studies that led to a first experimental trial. Subsequent work on Project EX follows. It is hoped that this cumulative effort will serve as a role model for more systematic tobacco use cessation research by others.

### Reasons for not quitting tobacco use

Youth report several of the same reasons for difficulty quitting tobacco use as adults. First, psychosocial variables are theorized to be relatively important reasons for not quitting and for relapse. Youth who report receiving more cigarette offers and who report having friends who smoke have been found to be less likely to quit smoking [5,6]. Skills to learn how to avoid or cope with tobacco use situations have been thought to be of great importance among adolescents [7-9].

Second, tobacco use dependence has been hypothesized as delaying attempts at quitting. Over half of adolescent smokers report having experienced withdrawal symptoms, an indication of level of addiction/dependence on nicotine. Reports of withdrawal symptoms among adolescents include craving, irritability, insomnia, hunger, and difficulty concentrating [5,6,10]. Information on addiction, withdrawal symptoms, strategies of quitting (e.g., tapering or abrupt withdrawal, substitutes), and coping with urges to use (e.g., "waiting it out," "seeing a slip all the way through") have been considered imperative to facilitate successful quit attempts [5-8].

If one is motivated to quit tobacco use, learning how to counteract both psychosocial reasons to continue using and withdrawal symptoms is quite important. However, most adolescent tobacco users are not motivated to quit in the next 30 days [4]. Thus, another reason youth do not quit (or relapse) includes a general lack of motivation to quit now as opposed to the future.

### Need to consider motivation

While many theories have addressed attitudes about tobacco use, most do not consider sufficiently the importance of one's motivation to learn new skills and follow through with quit attempts [11,12]. While most research on changing motivation to improve health has been conducted with adults, work has begun with teens and young adults as well, particularly in the arenas of teen drug abuse cessation [11,12] and cigarette smoking cessation [13,14]. There are at least two components of motivation to quit tobacco use: direction/goal and effort/energy [13]. First, tobacco users consider "why" they should not continue to engage in tobacco use (direction). As noted by Miller and Rollnick [12], "a discrepancy [made explicit] between present behavior and important goals will motivate change" (p. 58). Faced with salient discrepancies, the person may change his/her behavior to be better able to achieve desired goals. For example, a teen smoker may realize, or be educated to realize, that good health is important to help one maximize one's performance in school, that smoking is interfering with good health, and that one may need to quit smoking to help increase school performance.

The second component of motivation involves considering how much effort or energy youth are willing to expend to change their behavior. While unattained goals tend to reflect discrepancies between perceptions of possible and present behaviors or events, the energy component reflects sources of "pushes" or "pulls" to achieve the different goals. As examples, one may become aware of pressure from family, friends, or future employers to quit smoking. Also, one may feel capable, or be educated to feel capable, of sustaining subjective discomfort of nicotine withdrawal symptoms.

### History of teen tobacco use cessation research

Sussman [9] provided a review of the last two and a half decades of research in adolescent and young-adult tobacco use cessation. An exhaustive review was conducted of a total of 66 tobacco cessation intervention studies-targeted or population-published between 1975 and January 2001. Fifty studies were conducted in the United States, five in Canada, three in the United Kingdom, two in Australia, and one each in China, Finland, New Zealand, Norway, Sweden, and Switzerland. Six of these 66 studies were conducted in the 1970s, 15 were conducted in the 1980s, 43 were conducted in the 1990s, and two were conducted in 2000–2001. Thus, teen tobacco use cessation research seems to have become a more active research arena beginning in the 1990s.

Average reach and retention across the intervention studies was 61% and 78%, respectively, and was higher when whole natural units were treated (e.g., classrooms), than when units created specifically for the program were treated (e.g., school-based clinics). The mean quit-rate at a three- to 12-month average follow-up among the program conditions was 12%, compared to approximately 7% across control groups. A comparison of intervention theories revealed that motivation enhancement (19%) and contingency-based reinforcement (16%) programs showed higher quit-rates than the overall intervention cessation mean. Regarding modalities (channels) of change, classroom-based programs showed the highest quit rates (17%). Computer-based (expert system) programs also showed promise (13% quit-rate), as did school-based clinics (12%).

This study also examined 17 prospective survey studies of self-initiated cessation among young people. Key predictors of quitting were living in a social milieu that is composed of fewer smokers, less pharmacological or psychological dependence on smoking, anti-tobacco beliefs (e.g., that society should step in to place controls on smoking) and feeling relatively hopeful about life. Considered across the formalized intervention and self-initiated tobacco use cessation studies, key variables relevant to the quitting process include structuring the social environmental context for youth to facilitate quit efforts, motivating quit attempts and reducing ambivalence about quitting, and making the quitting process as enjoyable as possible, or minimizing the discomfort of sustaining a quit effort.

Engagement in a programmatic teen tobacco cessation process has been needed but hardly ever attempted. Project EX represented such an effort. Project EX was constructed on the foundation of a previous project, Project TNT, funded by the National Cancer Institute from 1987–1993. The first EX project began in 1997, followed by three subsequent EX projects. EX-1 was a school-based clinic experimental trial in southern California continuation (alternative) high schools [14]. As reported below and in other recent work [9,15,16], the results of EX-1 were very promising. Mediation work indicates that manipulation of motivation was a major determinant of quitting behavior in EX-1 [17]. EX-2 explored the generalizability of EX-1 findings to teens residing in Wuhan, China, using a single-group multiple baseline design [18]. EX-3 examined the incremental value of adding a pharmacologic adjunct to the EX clinic curriculum. Finally, EX-4 is examining the adaptation of the program to the regular classroom setting, as a prevention-cessation education curriculum.

### The Project TNT cessation trial

Project EX builds on the work of an earlier tobacco cessation project, Project Towards No Tobacco Use (Project TNT). Project TNT provided a state-of-the-art curriculum that was used as the template for the Project EX curriculum. Our objective was to enhance the standard curriculum by producing a program specifically tailored to motivate youth to quit smoking now rather than waiting until later [7].

Project TNT tested the efficacy of two tobacco use-cessation clinic programs within regular high school settings. The two curricula were similar in format (number of sessions; general type of session; and order of those sessions, such as introductory and preparation, followed by quitting), but one focused on the chemical dependency aspects of tobacco use while the other focused on psychosocial dependency associated with tobacco use. Cessation development involved small-scale assessments in Year 1 [19], recruitment development in Year 2, cessation clinic development in Year 3, and cessation intervention implementation and three-month follow-up in Year 4 (the main trial).

In the main trial, school-wide trends as well as clinic data were examined. Recruitment was achieved through flyers, public announcements, a sign-up list, person-to-person discussion, and class release time. The trial used an experimental design, multiple measures of cessation, multiple types of control groups, and multiple measurement time points. One control group was created by recruiting a group of would-be clinic participants and placing them on a wait list for the duration of the first clinic round. These students compose a motivated group, very similar to the program group. The other control group was composed of subjects who attended the same and other schools not involved in programming to examine school-wide trends.

Three-month follow-up data was collected (n = 244.) The results initially indicated approximately 20% versus 10% smoking cessation, and 44% versus 0% smokeless tobacco cessation, for clinic participants versus wait-list controls at three months post-clinic. Quit rates were then corrected by controlling for those who dropped out after the first clinic session (intent-to-treat analysis; i.e., assumption that drop-outs did not quit tobacco use) and use of biochemical validation to adjust for over-reporting of abstinence (saliva cotinine data was collected and analyzed). After these corrections, the data revealed no difference in clinic versus wait-list smoking cessation rate (about 7% cessation in each group). However, 13% smokeless tobacco use cessation was observed in the clinics and none was observed among the wait-list controls [7]. Also, school-wide trends for non-participants at program schools or control schools were in a direction showing an overall increase in tobacco use.

Through subsequent work, a five-session clinic was refined from the Project TNT clinic programs. Some clinic material was added (e.g., anger management) or combined (addiction and psychosocial dependency quit strategies), and nongeneralizable, relatively expensive program elements such as TNT key chains and calendars were eliminated. The final product of this process was the Project TNT comprehensive adolescent tobacco use cessation guide, a five-session combined curriculum, including both psychosocial and chemical dependency oriented aspects.

This combined program became the foundation for building a motivation-enhanced curriculum known as Project EX. Project TNT includes most standard tobacco cessation material, including information on reasons for using tobacco, physiological effects of tobacco use, nicotine withdrawal symptoms and strategies for managing them, psychological coping such as anger management and relaxation, avoiding weight gain, relapse prevention strategies, and maintenance of cessation through health promotion (e.g., exercise and dietary change).

## Project EX-1: a New School-based Clinic Program

Project EX-1 was a three-year project funded by the Tobacco-Related Disease Research Program (1997–2000). The approach utilized in Project EX-1 was to strengthen the scientific foundation and practical utility of a multi-session program using iterative development and evaluation [20].

### Project EX-1 program development studies

#### Focus Groups

Focus group methodology is one of the most widely used qualitative research tools in the applied social sciences [14,21]. This approach has been described as being applicable to various aspects of research including exploratory or hypothesis generation, clinical uses (assessing respondents' nonverbal as well as verbal behavior), phenomenological uses (generating data within a group process-oriented setting), and confirmatory uses (interpreting results obtained through quantitative methods).

Funded by an American Cancer Society seed money grant (1997–1999), an iterative focus group protocol was used to try to generate information to motivate youth to remain in a cessation clinic program, try to quit, and sustain a successful quit effort. Nineteen groups (total n = 233) composed of half tobacco users and half nonusers met for a 30-minute period to generate themes that might motivate youth to quit during the course of a cessation clinic. Each set of questions in subsequent rounds was developed derived from responses to questions in prior rounds.

Two types of focus group questions were asked. The first set of questions requested adolescents' reasons for smoking and quitting, and perceived efficacious activities. The second set focused on the drive-energy model of motivation. Youth reported that a trusting and intimate school-based quit-clinic environment was desired. One key obstacle noted was the challenge of figuring out how to avoid associating with other tobacco users. Keeping one's mind on something other than tobacco use was considered of major importance for quitting.

Numerous rewards of quitting were discussed among participants. These included: feeling better emotionally, receiving more social support after quitting, saving money, and feeling more respected by others/accomplished. Also discussed as rewards were having more energy; feeling less stressed; having fewer or no mood swings; having higher self-esteem; achieving more self-control; appearing cleaner, more attractive, healthier; being able to live longer and be more active; and achieving greater peace of mind.

Several of the focus group discussions led to activity development. The suggestion of use of nonsmoking/healthy activities and mention of yoga led to the development of a theme study activity, which was studied in the next stage of Project EX program development. The focus group participants saw mood as improving in a number of positive ways six months after quitting. This notion led to creation of another theme study activity that illustrates mood improvement at different time points after quitting. Ex-smokers were perceived as strong individuals; that influenced development of yet another theme study activity. This focus group study is described in more detail elsewhere [14].

#### Theme Study

A theme study consists of brief written descriptions of several hypothetical activities that are rated by students in the target population for the purpose of determining their potential interest, likeability, and perceived helpfulness. The theme study is an important step in the process of empirical program development because it allows the a priori ascertainment of subject preferences regarding potentially applicable activities. A theme study elicits information regarding the appropriateness of using certain topics in specific populations. The activities that yield high subject preference may then confidently be developed further into complete activities or sessions for immediate impact testing through component and pilot studies [23].

As an EX-1 program development study, the goals were twofold. First, there was an interest to determine which among two alternative presentation modalities, "game" or "talk show," of nine "traditional motivation enhanced" smoking cessation themes [13] were most acceptable to older teens. The game-type activities were those that involved team competition to learn material (e.g., board games). The talk show-type activities were those that involved a group facilitator, guests, and audience members to replicate such shows as "Oprah" while learning material. Second, there was an interest to determine which in a set of eight "novel" activities, derived from eight unique alternative-medicine-related themes, were as acceptable or more acceptable to continuation high school (CHS) students than the best activities derived from traditional motivation themes. A total of 26 activities were contrasted (total n = 391, half tobacco users), with the use of randomized orders of activities for different groups of raters. Details on the study design are provided elsewhere [24].

Talk show and game modalities were equally liked. The most highly rated activity was a talk show that emphasized quitting while one is young. Other highly rated activities tapped romantic choices when being tobacco free and not being a victim of tobacco company advertisements. Instruction in yoga also was preferred [24].

#### Component Study

A component study protocol involves actually exposing youth to an activity or session for its effects on measures of immediate impact [20]. The products of this type of study are the building blocks of a complete tobacco use cessation program. A total of 327 students participated in the EX-1 component study [25]. About half of the surveyed students were self-reported smokers, and these students were the focus of the analysis. The 14 most favorably rated activities from the theme study were retained for testing in the component study.

In this study, complete activity lessons were developed and immediate impact was assessed. Four activities were talk show type, five were game-type, and five were novel, alternative medicine-type. Activity sets were randomly assigned across classrooms such that each student was exposed to five activities over a three-day sequence [25]. The eight highest rated activities from the component study were joined with the standard five-session curriculum derived from Project TNT.

The top ranked activity was the game "Is Smoking on the Menu." In this activity, students create a menu of possible categories, and "order" (pick) questions regarding the dangers of passive smoke. The second highest ranked activity was a talk show that describes guests who are smokers at different stages of the quitting process. In this activity, the classroom facilitator (the adult health educator) serves as the show host. Several students volunteer to serve as guests for the show. They enact roles, based on information cards provided to them. The remaining youth serve as the audience, and are provided with a list of potential questions to ask the guests about tobacco use cessation.

The third highest ranked activity was yoga, a novel activity in which students learn several easy postures that they can use to feel more relaxed. The fourth highest ranked activity depicted family and friends confronting the smoker about smoking in a talk show format. The smoker talks about being nagged, whereas the family express their worries and how the smoker has become more irritable after becoming a smoker. The fifth highest ranked activity was about letting feelings pass. In this novel activity, participants learn that sometimes just letting feelings pass can be more effective than reacting to them. They also learn relaxation and breathing meditations. In the sixth highest ranked activity, a talk show, youth learn that younger quitters have an easier time than do older quitters in terms of tobacco use consequences. In the seventh highest ranked activity, talk show guests include an ex-smoker, a psychologist and a physician. Guests discuss how tobacco use actually increases, rather than decreases, stress. The final activity was a novel type that instructs on how smoking hurts one's breathing, and provides exercises on healthy breathing [25].

One activity, related to peer-motivational counseling, was rated highly in the theme study; it involved a presentation followed by a question-and-answer period led by a student who had quit smoking and could share his or her experiences with peers. This activity was dropped from the menu of activities after component testing at the first school because recruitment and retention of teen ex-smokers was difficult, and would be very difficult to institutionalize once a cessation program was developed [25].

#### Pilot Studies

Two rounds of pilot testing were conducted, in which recruitment, a school-wide pretest, an entire eight-session clinic, and a school-wide posttest were completed. Data points were developed and refined, including measures of regular tobacco use, consideration of measures of reach, retention, percent quitters and percent reduction [26]. In the first round, students were recruited to sign up for a tobacco use cessation clinic at one school and they participated in an eight-session program with no follow-up. The clinic was fine-tuned on the basis of the first round. In the second round, the clinic was tested along with a school-as-community component at a second school. Both components were fine-tuned after that round.

The "school-as-community" component was modeled on a drug abuse prevention project component [27]. The Associated Student Body, under teacher supervision, organized service, recreational, and job training functions and, through a project newsletter, permitted expression of anti-tobacco use attitudes at the school. Results at the two pilot schools were nearly identical. The school enrollments were approximately n = 125. A total of 100 students received a pretest at each school, and 25 youth were enrolled in each clinic. Any use of tobacco in the last 30 days was the behavioral inclusion criterion used. Across the two pilots, the reach was approximately 53% of all tobacco users. Retention at the clinics was approximately 60%. The percent quitters (of all tobacco products) were approximately 16% of those who attended the first session (biochemical validation was completed through CO analysis).

### Project EX-1 main trial

Project EX-1 involved development and implementation of a motivation-enhanced school-based cessation clinic program, compared to a condition which also included a school-as-community component in addition to the motivation-enhanced clinic, and a standard care condition, as a three-condition true experimental field design with 6 schools per condition. We hypothesized that a school-as-community component (primarily interpersonal motivation) together with a motivation-enhanced cessation clinic (primarily in-trapersonal motivation) may lead to higher cessation rates than use of a clinic alone because events in the nearby social climate influence quit efforts.

#### Program Intervention

Students engaged in a series of interactive activities assigned to influence motivation, self-concept, and efficacy. The first session served as an introduction to the clinic by setting ground rules and presenting an overview of the coming activities. In addition, reasons to quit were discussed by way of a talk show "Friends and Family Confront Smokers About their Habit," in which participants were asked to role-play various characters. In one situation, a "smoker" expressed being nagged by family and friends about his/her smoking; family and friends expressed their worries about the smoker's health and distancing issues that have arisen since the smoking began.

The relationship between stress and smoking was discussed in the second session by way of another talk show, "Your Cigarettes May Be Stressing You Out." Misperceptions regarding the effectiveness of cigarettes in relieving stress were addressed (e.g., the damaging effects of smoking on breathing, the importance of healthy respiration in stress relief). Students also practiced various diaphragmatic breathing techniques, a relatively novel activity.

The harmful substances contained in tobacco were reviewed in the third session along with the damaging effects these substances may have on the human body. Students played a tobacco trivia game called "Is Smoking on the Menu?," where participants "order" (pick) from a menu of dangerous passive smoking consequences categories and receive points for answering questions within the chosen category correctly.

The first steps to breaking an addiction were presented in the fourth session. Students were encouraged to make a commitment to quit using tobacco and various means of quitting were discussed. In addition, physical and psychological aspects of withdrawal were presented. Finally, a talk show, "Quitting Smoking: I've Been There and It Does Get Better," was conducted.

Session five discussed further the addictive properties of nicotine and presented more strategies for managing withdrawal symptoms. Psychological coping strategies including self-forgiveness and avoiding false expectations were reviewed.

Quitting maintenance strategies were presented in the sixth session. In addition, students practiced a "yoga activity," in which several easy postures are learned to promote a sense of balance and relaxation.

Session seven involved learning additional quitting maintenance strategies including anger management and assertiveness training. A novel meditation activity called "Letting Feelings Pass" was performed while imagery and visualizations were prompted. Students learned that sometimes just letting feelings pass can be more effective than immediately reacting to them.

Finally, the eighth session involved learning means to avoid relapse. Students were also motivated to quit now rather than later, through a talk show, "Warning: Waiting to Quit Smoking May Be Hazardous to Your Peace of Mind." The focus of this talk show was that it is better to quit smoking and sustain the quit attempt while a person is young, due to the accumulation of consequences accompanying age.

#### Recruitment, Subjects, and Design

This program was developed and the main trial study was implemented at continuation high schools, the alternative high school system in California. Continuation high schools were established in 1919 pursuant to the California Educational Code (Section 48400), which requires continued (part-time) education for all California youth until reaching age 18. Usually students who are experiencing life difficulties when beginning comprehensive (regular, traditional) high school transfer to continuation high schools where hours are more flexible and the teacher:student ratio is twice as high as at comprehensive schools (1:15 vs. 1:30). Every school district that has an enrollment of more than 100 students in 12th grade must have a continuation school program; there are over 600 continuation high schools in California. Continuation high school students report much higher levels of cigarette smoking (but not smokeless tobacco use) than traditional high school students. For example, smoking at least weekly on a current use measure is approximately 47% versus 15%, respectively, at mean age = 16.5 years [26].

Recruitment included use of flyers, brief public announcements in each classroom, word of mouth, and class release time. A total of 260 youths at 12 schools were enrolled in clinics, and 70 smokers were identified and surveyed at six control schools. Thus, 330 youths were included in this design, making this the largest single experimental teen smoking cessation trial conducted up to that time. A total of 64% of the sample were male; 47% were Latino, 27% were white, 8% were Asian, 6% were African American, and 12% were "other." The mean age was 16.8 (SD = 0.8), with a range from 14–19 years of age. The demographics of the clinic enrollees did not differ from the general population of smokers at these schools.

The school-as-community manipulation was successful. Tobacco use-focused Associated Student Body meetings were held at each of the school-as-community schools for an average of five months, involving 6% of the students at the school. Six tobacco-free or anti-smoking events were held at each of these schools, which involved an average of approximately 20% of the student body per event, and there were a greater number of anti-tobacco use-focused activities at these schools than at the other 12 schools involved in this trial. Follow-up involved telephone calls to smoker groups beginning three-months post program. A four-month window was permitted to try to reach youth. For those who reported quitting, biochemical validation was used to confirm reports (CO data collection).

A total of 34% of the target population of tobacco users enrolled in the clinics. All subjects smoked cigarettes; 46% smoked only cigarettes, 6% both smoked cigarettes and used smokeless tobacco, 36% smoked both cigarettes and cigars, and 12% used all three tobacco products. Among these cigarette smokers, 85% smoked daily with an average of 8.8 cigarettes per day (SD = 9.3) As scored on Prokhorov and colleagues' [28] adaptation of the Fagerstrom, 30% scored in a light addiction range (0–6), 53% scored in a moderate addiction range (7–13), and 17% scored in a heavy addiction range (14–21). A total of 142 smokers completed the clinic for a retention rate of 55%.

All sessions were rated as being "very helpful" by students and schoolteachers. There were no differences in outcomes between the clinic plus school-as-community condition and the clinic-only condition; thus, data were merged across these two groups. The intent-to-treat, biochemical validation-adjusted quit rate at the end of the clinic was 14%. A total of 60% of the program enrollees were reached at follow-up, which occurred an average of 3.7 months (SD = 0.7 months) after the posttest (approximately five months after the clinic "quit day" in Session 4). Also, 70 smokers were consented to be followed up from the control schools, and 42 were reached at follow-up (60%). The intent-to-treat, biochemical validation-adjusted follow-up 30-day quit rate (of all tobacco products) was 17% across all program condition enrollees. The quit rate (of all tobacco products) among the control school smokers at follow-up was 8%. The odds ratio = 2.36, p < .05, intent to treat model, with correction for biochemical validation measurement [29].

#### Mediation of Project EX-1 Program Effects

Project EX-1 aimed, in part, to enhance students' motivation to quit the use of tobacco. A subsequent secondary analysis study attempted to ascertain whether or not the program did, indeed, manipulate motivation, and whether motivation mediated program effects. A total of 168 student tobacco users from the EX-1 trial were surveyed at all three time points, which provided the sample on which to engage in a mediation study. Also, measures were developed to tap mediation effects. Motivation to quit was measured with 16 four-point items. An eight-item energy index (Cronbach coefficient alpha = .71) measured areas such as, "I feel a lot of pressure from my friends and family to quit my tobacco use," and "How much energy do you have to quit tobacco now and/or stay stopped?" An eight-item direction index (Cronbach coefficient alpha = .81) included items such as, "How much direction do you feel you are receiving to quit tobacco now and/or stay stopped?," and "Quitting tobacco use improves one's mood." Item responses ranged from either "a. A lot" to "d. None" or "a. Strongly agree" to "d. Strongly disagree."

Mediation analysis as recommended by Barron and Kenny [30] was employed. Both direction (F = 60.63, p < .01) and energy (F = 41.98, p < .01) were significantly related to the treatment condition while controlling for pre-motivation differences. Also, program effects were replicated here by way of a regression model. Another essential question is whether post-treatment motivation, alone, predicted quit status at follow-up. Reported levels of energy at posttest significantly predicted non-use of tobacco in the last 30 days (F = 7.41, p < .05). Direction was also a significant predictor of abstention from tobacco use in the last 30 days (F = 14.58, p < .01).

A final regression model was used to determine if treatment effects were mediated by changes in energy, direction or both. Again, upon entering the mediator variables into the regression model, a reduction in the size of treatment effect should occur. The standardized estimate for the treatment clinic was reduced from .17 to .09 when direction and energy effects were added. Indirect effects via energy were determined by taking the product of standardized estimates from the effect of treatment on post energy (path a) and from the post energy effects on quitting (path b). Thus, a total effect size for energy was calculated at .03. In conjunction, indirect effects through the direction index were calculated to be .03. Because direction and energy were correlated (r = .82), total effect sizes for direction and energy were summed and then divided by the total effect size for treatment. Thirty-eight percent of the treatment effect can be accounted for by motivation components. Fifty-three percent of the treatment effects remained unaccounted for and 8 percent was due to reported motivation levels at pretest [17].

The findings of the current study suggest that teen tobacco use cessation programs would benefit from a motivational component to quit now rather than later. Most behavior has multiple influences, and from a psychosocial perspective, a significant reduction in the effects of an independent variable on a dependent variable due to mediation is regarded as an indication of a potent mediator. Further studies need be conducted to examine specifically which activities influence which motivational components. In addition, the potency of the influencing activities must be measured.

### Project EX-2: The Wuhan Pilot Study

To examine effectiveness and receptivity of Project EX in another culture, where motivations for smoking and quitting may be different from those in the United States, and to provide more information for developing smoking cessation programs for Chinese adolescent smokers, a pilot test of Project EX was conducted in Wuhan, China, in 2001. The increasing smoking prevalence in Chinese adolescents in China indicates a need for effective smoking cessation programs, to stop the habit before physical consequences accumulate [31,32].

#### Study development

Wuhan is a large city in Central China, with a population of 7.3 million and an area of 8,467 square kilometers. Located on the Yangtze River, Wuhan is an economic, cultural, and political center of China. There was an ongoing prevention trial conducted among Chinese adolescents in Wuhan in 2000 by the University of Southern California (USC) and Wuhan Public Health and Anti-epidemic Station. Results of the prevention trial showed that smoking prevalence among adolescents in Wuhan is high. Lifetime smoking prevalence was 47% among boys and 18% among girls; 16% of boys and 4% of girls were current smokers [32]. The utility of teen tobacco use cessation was explored as part of, and in addition to, this other work being conducted.

#### Curriculum

The EX-1 curriculum was translated to Chinese (Mandarin) by two certified translators, who were employed by USC. Two bilingual researchers at USC verified the translation by reading both the English and Chinese versions. The translated version was pilot-tested session-by-session in focus groups at the Wuhan Public Health and Antiepidemic Station before program implementation to verify that the version to be utilized was both clearly understood and culturally appropriate. Only six minor changes were made in the curriculum to adapt it to Wuhan culture, as is described elsewhere [18].

#### Methods and Results

Participants were 46 10th graders in Wuhan who attended either a regular high school (51%) or a vocational school (49%). The mean age of the study participants was 16.2 (SD = 0.4) years, with a range from 16 to 17 years. Among the participants, three were girls. All participants were of Han ethnicity. The 46 participants were recruited from a school-based screening assessment conducted in June 2000, two and one-half weeks prior to administration of the baseline clinic assessment. The screening questionnaire was completed by 622 10th grade students. The two and one-half weeks that passed between the initial assessment with the announcement about the availability of the quit-clinic, and the first clinic session, permitted a means of estimating naturally occurring cessation among youth from the same clinic cohort. A total of two of the 68 participants originally screened reported having quit during this period. Thus, naturally occurring quitting was estimated at 3%.

Paper-and-pencil pretest and posttest questionnaires were administered in the beginning of the first and end of the last clinic sessions. A follow-up assessment was conducted approximately four months after the last session of the clinic. Saliva samples were collected at the beginning of each session and at the follow-up, utilizing a pipeline protocol [7].

At the baseline, the participants reported smoking a mean of 5.7 cigarettes per day. Forty-five of the 46 participants attended at least six of the clinic sessions and completed the immediate posttest questionnaire (98% of the initial participants). All 45 of these participants also completed the follow-up questionnaire, a mean of 4.6 months after the posttest (SD = 0.9 month).

The eight process ratings (e.g., helpful, interesting, liked) were highly inter-correlated (coefficient alpha = .85). These eight process adjectives were averaged to compose an index; the mean score on this index was 8.10 (SD = 1.07). All eight individual clinical sessions were rated as high in quality (all session rating means > 7.4).

A saliva sample (about 5 ml) was collected. The cotinine levels of the saliva samples were assessed using the NicoMeter strip, an immunoassay-based analytical technique. An over-reporting of quitting was observed in 4.5% of the sample based on the results of biochemical validation. Adjusting our results downward for this bias, then, at the four-month follow-up, 10.5% adjusted 30-day abstinence and 14.3% adjusted past-week abstinence are reported. This is 3.5–4.8 times the quit-rate achieved prior to the beginning of the clinic. Since the same bias is likely to have occurred among those reporting naturally occurring quitting, the same apparent benefit of attending the clinic would be achieved.

This study provided evidence that Project EX, an adolescent smoking cessation program developed for use in the United States, also may be effective among adolescents in China. The results of this study indicated that it is feasible to implement smoking cessation programs among Chinese adolescents, that Chinese adolescent smokers are receptive to cessation programs such as Project EX, and that this program is effective in increasing short-term smoking cessation rates. These results suggest an optimistic future for use of Project EX in China.

### Project EX-3: Nicotine Gum and Herb

While many researchers and practitioners have suggested use of pharmacological adjuncts with youth, only two published studies appeared in the literature as of 2002 [9]. These studies failed to show an advantage of the use of nicotine replacement therapy with youth. The research objective of the present study was to provide a scientifically rigorous evaluation of the effectiveness of a school-based tobacco use cessation program utilizing three features. The first feature included a comprehensive on-site promotion for program enrollment based on school organizational theory and practice. An on-site program promotion and recruitment protocol was developed and finalized. The second feature was to develop an on-site, multi-session, behavioral intervention program. The content of the Project EX school-based clinic program (EX-1) was modified to accommodate the use of tobacco substitutes (gum), including expanding the topics of nicotine dependence and addiction, careful screening of eligibility for tobacco substitutes, detailed coverage on the use of the substitutes, and in-school gum distributions.

The third feature was a randomized, open label trial of adjunctive nicotine replacement versus substitution therapy, comparing nicotine-and non-nicotine-containing gum in a two-condition experimental design. Nicotine gum (polacrilex) was approved by the FDA in 1984 and was the first nicotine replacement therapy available to nicotine addicts. The gum contains a nicotine resin complex in a buffered chewing-gum base. Nicotine dosages of either 2 or 4 milligrams (mgs) are released after 30 minutes of normal chewing [33]. A meta-analysis of 46 trials evaluating nicotine gum in adult cessation studies, with a combined total of 1,273 patients, revealed a quit rate of 19% among those who received gum at 12-month follow-up compared to 11% among 966 patients who did not receive gum (odds ratio = 1.89) [34]. This compares to 12-month quit-rates of 16% for use of nicotine patches (20 studies). Clearly nicotine gum is as effective as nicotine patches as an aid to adult cessation. The highest quit rates with NRT have been obtained when it is used in combination with behavioral counseling. Meta-analysis on smoking cessation trials that used gum and were accompanied by behavioral interventions revealed an average odds ratio for abstinence among users of the patch of 2.07 (over 46 studies) at six month follow-ups [34].

#### Method and Results

Coordinated through the County Office of Education, in cooperation with individual school health education staff and school nurses, all high school students and staff in Humboldt County received in-school comprehensive program promotion sessions. These sessions focused on increasing awareness of in-school availability of support for quit-efforts and motivation to quit and/or cessation support strategies. Students who smoked and desired to quit were further screened for research study eligibility regarding nicotine replacement/substitution therapy at clinic intake sessions. Sixteen high schools in Humboldt County were the primary study sites. Interested tobacco-using students were contacted and screened for eligibility. To be eligible for the program, potential subjects had to: 1) have used tobacco at least 100 times in their lifetime, and 2) be currently using tobacco at least once per week. In addition, subjects 3) could not be pregnant or planning on pregnancy (girls), 4) were physically healthy (based on a brief physical exam and medical history review), 5) showed a commitment to quit using all tobacco while taking the study drugs, and 5) provided signed parental consent and self-assent. Subjects that did not qualify for the drug study were allowed to participate in a quit-clinic at no cost.

Those entering the study (total subject N = 117) were randomly assigned to receive either nicotine gum (n = 57) or a herbal gum control (n = 60). Subjects were 66% female, 72% white, 12% Native American, and 15% other ethnicity, and all lived in rural areas. Also, 59% of students were from regular high schools, and 41% were from alternative high schools. Subjects smoked a mean of 10.2 cigarettes per day at baseline (SD = 6.6). In addition, 6% of subjects also reported use of smokeless tobacco at baseline. An open label, nicotine substitution gum controlled design was used and is among the most practical for this context because it provides 1) a look- and use-alike medication without the active ingredient thought to be the mechanism of action (nicotine), and 2) an expected efficacy control.

A fairly detailed self-report questionnaire was administered to all students who attended the clinic intake session. Subsequent assessments (at posttest, and two and six months follow-up) included self-reports of withdrawal symptoms, tobacco use behavior (slips, current use level), use of and problems with the gum, treatment self-efficacy and outcome efficacy, and attitudes towards features of the clinic (ratings of the session and materials provided). Subjects who reported non-use of tobacco in the last seven days were tested at the two and six month follow-ups after they completed their self-reports of use and were administered saliva cotinine to validate those self-reports.

The first five sessions were held in a one-week period, followed by three sessions that were held once every two weeks. The first four sessions were the same for all participants. The nicotine replacement/substitution phase of the study, for those who were eligible, began at Session 5. The timing of the subsequent session coincided with the six-week gum treatment protocol, and effectively provided for monitoring and distribution of the gum supply in "manageable" doses. A registered school nurse or physician assistant was available at the drug distribution sessions and assisted in delivery of the study drug protocol.

This study used the Nicorette^© ^Nicotine gum (polacrilex) as the "active" nicotine replacement delivery modality. Plasma nicotine concentrations from a single 2 mg piece are approximately one-half to one-third those delivered by a single cigarette over a one-hour period in adults [35]. The study also used a nicotine substitute, CigArrest gum, which is a non-nicotine containing herbal gum. Both types of gum were distributed on the same schedule with similar instructions for use. The study drugs (gums) were used for a six-week period, between clinic Session 5 and Session 8.

At the first distribution session (clinic Session 5), subjects who registered positive for cotinine were given enough gum to completely substitute one piece of gum for every tobacco product use for a two week period, up to a maximum of 12 pieces per day (168 pieces). This was the 100% substitution phase. The second allocation of gum (at Session 6) was 66% of the gum used in the between sessions 5 and 6. Similarly, the third (and final, Session 7) distribution was 50% of that used between sessions 6 and 7, with a maximum of one-third their original allocation.

Subjects were comparable across the two conditions at baseline. Seventy-five percent of subjects completed the sessions and 87% were followed-up six-months post-program. At two-months follow-up, the intent-to-treat 30-day quit rates for all tobacco products were 11% in the Nicorette condition and 13% in the CigArrest condition. At six-months follow-up, the intent-to-treat 30-day quit-rates were 16% in the Nicorette condition and 15% in the CigArrest condition.

### Project EX-4: Classroom Based Prevention-Cessation Program

Project EX thus far is being delivered as a school-based clinic. While this school-based clinic version of Project EX is effective, its reach is limited to those who attend the clinic. Both tobacco users and nonusers are found in the classroom setting. However, there are four reasons to bring cessation education material into the continuation high school classroom setting. First, continuation high school youth are at very high risk for regular tobacco use. A total of 85% of these youth have tried tobacco, 71% have an intention to try tobacco in the future or are monthly users, 57% are monthly users, and 48% are daily users. Among the monthly users, approximately 50% only smoke cigarettes, whereas 50% smoked cigarettes and use another tobacco product as well (mostly cigars). Youth at CHSs who do not smoke are confronted with smoking among their peers on a daily basis. This would seem an appropriate context to reach all its youth with tobacco use education programming. Second, this modality greatly increases the reach of programming and, therefore, may increase the total numbers of quitters.

Third, such programming can be framed so as to exert a preventive function among youth who are not current tobacco users. Comprehensive social influence programming is relatively unlikely to be effective for older, high-risk teens [6]. Motivation enhancement, social skills, and life skills material, contained in Project EX, is likely to be more relevant. In fact, program development studies for Project EX took place in the CHS classroom setting, composed of users and nonusers. The nonusers enjoyed the programming as much as, or more than, the tobacco users. Also, they perceived that the activities developed would help them to not use tobacco in the future.

Finally, cessation as classroom programming can be a useful practical health science educational tool in the high school classroom setting. Tobacco use addiction and cessation is an important topic to be instructed by the time a youth graduates high school, because of intrinsic academic importance in health behavior science and to appreciate the practical, societal costs of addiction.

This newest study has three main aims. The first goal has been to adapt Project EX clinic program to the classroom context in continuation high schools (CHSs). Material has been edited and piloted in classrooms, and has been made appropriate as a means of cessation and indicated prevention. In EX-4, the prevention changes included adding experiments in Session 1 for non-smokers to try. Non-smokers choose one situation in which they are around people smoking tobacco and notice how they feel (e.g., does the second hand smoke bother them). Non-smokers who are never around smokers notice where any evidence of tobacco is (e.g., corner store, advertisements, cigarette butts on the street, etc.) and discuss how they feel about it. We also added reasons why youth should remain tobacco-free. In Session 2, information was added on tobacco industry marketing tactics and how they target youth. More information about secondhand smoke was added in Session 3. In Session 4 non-smokers can make personal commitments to remain tobacco-free or serve as a "listening ear" to assist those who may be trying to quit. In Session 5 non-smokers are given suggestions on what one should and should not do to serve as a "listening ear" to assist those who may be trying to quit. Also, non-smokers and smokers are given exercise tips and general information on food groups and serving sizes in Session 6.

The second goal has been to implement and assess effects of the classroom program at seven CHSs. This eight-session program is now being compared to a standard care control condition (classroom assessments only) at seven other CHSs, with immediate pretests, immediate posttests, six and 12-month follow-ups, in a two-group experimental design. We expect the quit rates achieved will be at least as high as those previously obtained with the Project EX clinic-based program. The overall number of youth who quit smoking will be higher in the classroom delivery context by reaching most tobacco users at the school. Also, we expect that those who do not report smoking in the last 30 days who are exposed to the classroom program will exhibit lower levels of smoking six and 12 months later compared to those in the standard care control condition.

Finally, we will develop a training of trainers manual, and offer one-day Project EX classroom training to two school personnel per school at all CHSs (n = 28). School personnel will be assisted and paid to provide the classroom program. We will monitor integrity of delivery and feasibility of program dissemination in the continuation high school classroom context.

The main hypothesis is that a classroom delivery of Project EX, which provides substantive material contents that appeals to both non-regular and regular adolescent tobacco users, will exert a greater impact on tobacco use measured six and twelve months later than will a standard care control condition. Only main effect differences (no interactions) are expected when comparing conditions. A separate analysis will be completed for the program's impact on smoking cessation among baseline smokers, and on smoking prevention among baseline non-smokers. In addition, we will assess among both smoker and non-smoker subjects the program's effect on slowing the progression from non-smoking or light smoking to heavier smoking, or on accelerating the progression from heavier smoking to lighter smoking. The end-point in such analysis is changes in a five-category tobacco involvement measure, which was developed to better describe the full range of tobacco use in the continuation high school classroom. We predict that with six-month as well as 12-month follow-ups, the mean tobacco involvement increase on this five-category tobacco involvement measure will be significantly less in the program group compared with the control group [36]. The outcomes for this study should be available in 2006.

## Discussion

Previously, our research group found a control group 30-day quit rate of 3% (EX-2) to 8% (EX-1). Our intent-to-treat program quit rates were 17% (EX-1), 14% (EX-2), and 16% (EX-3), which at least doubled that found in control comparisons in the two studies that provided them [14,18]. The fact that participants who dropped out of the program conditions were considered as continuing to smoke in analyses completed provided a conservative test of program efficacy. This program also showed promise of generalizability among high school youth in China [18] in a multiple baseline single group design. EX-3 showed that the effects could last over a six-month posttest follow-up for youth at both alternative and comprehensive high schools. Finally, EX-4 is testing the utility of EX in a classroom setting, as opposed to a school-based clinic, and will attempt to achieve effects on all youth along the tobacco non-use to use continuum.

These results are consistent and provide a strong case for EX. However, there are interpretational problems of the results of these studies. First, the Project EX trials used control groups that were not necessarily matched with the program groups on pretest motivation to change. Certainly, standard care controls are less motivated to quit tobacco use than are wait-list controls. Still, the EX-1 mediation study revealed that the program worked statistically controlling for pretest motivation for change [17]. Also, the multiple baseline design used in EX-2 involved subjects of similar levels of motivation to quit. Future studies might consider more use of wait-list control groups. Second, different means were used across studies of controlling for self-reports through biochemical validation. EX-1 and EX-4 have or are using CO readings, whereas the other studies used saliva cotinine testing. Still, results are consistent across trials after making adjustments for whatever biochemical validation protocol was used. Third, most of the data reported in these studies pertained to cigarette smoking. Less emphasis was placed on use of other tobacco products, which tended to be used by some youth in addition to cigarettes but not instead of cigarettes. While the cessation results pertained to use of all tobacco products, future research might pay additional attention to smokeless tobacco and cigar smoking, as examples.

As of the writing of this paper, EX has been selected as an evidence-based program by the Substance Abuse and Mental Health Services Administration (SAMSHA). The school-based clinic version soon will be disseminated nationwide, as one of only two model-teen tobacco use cessation programs. The other program is Not On Tobacco, or NOT [37]. While this is good testimony for the fruits of rigorous research, there will be much more work to be done once EX is disseminated. It is not known who will deliver EX in the field since only trained health educators have delivered it thus far. Teacher training is needed and may lead to appropriate use by teachers at schools who are trained similarly to our health educators. However, research has not yet shown that schoolteachers can successfully deliver EX.

Second, it is not known how long effects might last. At present, for example, it is not clear that effects would last for at least a year. Third, it is not clear where EX might work and where it might not work, though the data from Wuhan, China, is promising. Finally, it is not clear if the program will be delivered in its entirety by schools, and if youths' confidentiality as smokers will be able to be maintained. There is much future research to complete.

## Competing interests

The authors declare that they have no competing interests.
